# Enhanced CD8 T-cell anti-viral function and clinical disease in B7-H1-deficient mice requires CD4 T cells during encephalomyelitis

**DOI:** 10.1186/1742-2094-9-269

**Published:** 2012-12-14

**Authors:** Timothy W Phares, Stephen A Stohlman, David R Hinton, Cornelia C Bergmann

**Affiliations:** 1 Departments of Neurosciences NC30, Lerner Research Institute, Cleveland Clinic Foundation, 9500 Euclid Avenue, Cleveland, OH, 44195, USA; 2 Department of Pathology, Keck School of Medicine, University of Southern California, Los Angeles, CA, 90033, USA

**Keywords:** Central nervous system, Encephalomyelitis, CD4^+^ and CD8^+^ T cells, Gliatropic coronavirus, Inflammation, Axonal damage

## Abstract

**Background:**

Anti-viral CD8 T-cell activity is enhanced and prolonged by CD4 T-cell-mediated help, but negatively regulated by inhibitory B7-H1 interactions. During viral encephalomyelitis, the absence of CD4 T cells decreases CD8 T cell activity and impedes viral control in the central nervous system (CNS). By contrast, the absence of B7-H1 enhances CD8 T-cell function and accelerates viral control, but increases morbidity. However, the relative contribution of CD4 T cells to CD8 function in the CNS, in the absence of B7-H1, remains unclear.

**Methods:**

Wild-type (WT) and B7-H1^−/−^ mice were infected with a gliatropic coronavirus and CD4 T cells depleted to specifically block T helper function in the CNS. Flow cytometry and gene expression analysis of purified T-cell populations from lymph nodes and the CNS was used to directly monitor *ex vivo* T-cell effector function. The biological affects of altered T-cell responses were evaluated by analysis of viral control and spinal-cord pathology.

**Results:**

Increased anti-viral activity by CD8 T cells in the CNS of B7-H1^−/−^ mice was lost upon depletion of CD4 T cells; however, despite concomitant loss of viral control, the clinical disease was less severe. CD4 depletion in B7-H1^−/−^ mice also decreased inducible nitric oxide synthase expression by microglia and macrophages, consistent with decreased microglia/macrophage activation and reduced interferon (IFN)-γ. Enhanced production of IFN-γ, interleukin (IL)-10 and IL-21 mRNA was seen in CD4 T cells from infected B7-H1^−/−^ compared with WT mice, suggesting that over-activated CD4 T cells primarily contribute to the increased pathology.

**Conclusions:**

The local requirement of CD4 T-cell help for CD8 T-cell function is not overcome if B7-H1 inhibitory signals are lost. Moreover, the increased effector activity by CD8 T cells in the CNS of B7-H1^−/−^ mice is attributable not only to the absence of B7-H1 upregulation on major histocompatibility complex class I-presenting resident target cells, but also to enhanced local CD4 T-cell function. B7-H1-mediated restraint of CD4 T-cell activity is thus crucial to dampen both CD8 T-cell function and microglia/macrophage activation, thereby providing protection from T-cell-mediated bystander damage.

## Background

The magnitude, quality and longevity of CD8 T-cell effector function is positively regulated by CD4 T cells, and negatively regulated by various T-cell inhibitory molecules. CD4 T cells augment CD8 T-cell activation and expansion, directly through the production of cytokines or indirectly by licensing dendritic cells (DCs) in draining lymph nodes [1,2]. Moreover, CD4 T cells can further enhance the primary anti-viral responses of CD8 T cells and promote their survival in the target organ [3-8]. This function is especially crucial in sustaining CD8 T-cell activity during prolonged and chronic infections. Paradoxically however, both CD4 and CD8 T cells upregulate numerous inhibitory molecules upon extended exposure to antigen to counterbalance over-exuberant, and potentially damaging, T-cell activity. Negative regulation by T-cell engagement of inhibitory ligands allows customized fine-tuning of T-cell function and mobility by the respective antigen-presenting cells (APCs) in the local environment. Among the components regulating the delicate balance between protective and detrimental immunity is programmed death (PD)-1, which dampens T-cell proliferation, cytokine production, and cytolytic activity following interaction with its ligand B7-H1.

The ongoing regulation of T cells and their adaptation to the local environment is most apparent during persistent infections, when CD4 T cells are essential to prolong CD8 T-cell function and survival [9-11]; however interactions between inhibitory receptors and their ligands dampen anti-viral function [11-15]. This paradigm also applies to encephalomyelitis induced by the sub-lethal gliatropic JHM strain of mouse hepatitis virus (JHMV). In this model, T cells control acute virus replication using both perforin-mediated and interferon (IFN)-γ-mediated mechanisms [16-19]; however, CD8 T-cell function rapidly wanes, allowing persistent infection [20]. Furthermore, T-cell activity is associated with immune-mediated demyelination, which is sustained throughout the viral persistence [21]. CD8 T cells are primary adaptive anti-viral effectors, but CD4 T cells play a vital supportive role, and may also directly contribute to viral control [8,16-19]. Depletion of CD4 T cells at distinct times relative to infection showed that CD4 T cells not only enhance peripheral CD8 T-cell priming/expansion, but further promote CD8 T-cell function locally within the central nervous system (CNS) [8]. CD8 T cells deprived of CD4 T-cell help within the CNS (designated ‘unhelped’ CD8 T cells), have diminished effector function and are unable to control virus replication [8]. Recent analysis of the contribution of inhibitory interactions during JHMV pathogenesis further showed that virus-specific CD8 T cells in the CNS express high levels of PD-1 [22]. Moreover, oligodendroglia, which are prominent targets of infection, strongly upregulate the ligand B7-H1 in response to IFN-γ during infection [22]. In a previous study, the dampening effects of PD-1:B7-H1 interaction were evident by increased IFN-γ and granzyme B production by CNS-infiltrating CD8 T cells, coincident with accelerated virus control and decreased viral persistence in JHMV-infected B7-H1 deficient (B7-H1^−/−^) mice [15]. Conversely, however, the extent of axonal damage was exacerbated, suggesting that B7-H1 mediates protection from immune pathology and mortality [15]. Oligodendrocytes were shown to upregulate major histocompatibility complex (MHC) class I, but not MHC class II during JHMV infection [23], thus the improved viral control was attributed to enhanced CD8 T-cell activity. However, demyelination was similar to infected wild-type (WT) mice [23], suggesting that the absence of B7-H1 did not overtly increase the vulnerability of oligodendrocytes to immune attack. This conundrum led us to explore the possible contribution of CD4 T cells to enhanced viral control and pathology, especially as CD4 T cells are potent producers of IFN-γ *in vivo*, and are strongly associated with pathogenicity and clinical disease [4,24].

The current study characterizes the relative contributions of CD4 T-cell help and B7-H1 to CD8 T-cell effector function in the CNS during viral encephalomyelitis by addressing three interrelated questions. 1) Is enhanced CD8 effector T-cell activity and viral control in B7-H1^−/−^ mice dependent on local CD4 T cells, similar to WT mice? 2) Is CNS CD4 T-cell function dampened by B7-H1? 3) Is increased axonal damage in the absence of B7-H1 sustained in the absence of CD4 T cells? To ensure presence of CD4 T-cell help during priming but withdrawal of helper function within the CNS, CD4 T cells were depleted in B7-H1^−/−^ mice subsequent to the initial expansion phase, but before CNS entry [8]. Numbers and composition of CNS-infiltrating cells were comparable in CD4-depleted mice; however, decreased granzyme B and IFN-γ expression by CD8 T cells correlated with loss of viral control. Moreover increased CD8 function was associated with increased CD4 T-cell activity in B7-H1^−/−^ mice. The absence of CD4 T cells in B7-H1^−/−^ mice did not affect demyelination or significantly lessen axonal damage, but did improve clinical disease and survival.

## Methods

### Ethics approval

All procedures were conducted in accordance with animal protocols approved by the Institutional Animal Care and Use Committee.

### Mice, virus infection, and CD4 depletion

The WT mouse strain was C57BL/6 (National Cancer Institute (Frederick, MD, USA). B7-H1^−/−^ mice on a C57BL/6 background were previously described [25]. Mice were housed under pathogen-free conditions at an accredited facility in the Cleveland Clinic Lerner Research Institute. Mice were infected at 6–7 weeks of age by intracranial injection with 250 plaque-forming units (PFUs) of the gliatropic JHM variant V2.2-1 of mouse hepatitis virus (JHMV) [26]. Recipient animals were depleted of CD4 T cells by intraperitoneal injection with 250 μg of anti-CD4 (α-CD4) monoclonal antibody (mAb) GK1.5 at 4 and 6 days post-infection (p.i.). Control animals received the same amount of α-βgalactosidase (α-βgal) control mAb GL113. Recipients were depleted of CD8 T cells by intraperitoneal injection with 250 μg of anti-CD8 (α-CD8) mAb 2.43 at day −2, 0, and 7 p.i. These regimens resulted in more than 99% depletion of CD4 or CD8 T cells in the periphery and CNS. Control animals received the same amount of control mAb GL113. Animals were scored daily for clinical signs of disease on a four-point scale (0 = healthy; 1 = ruffled fur and hunched back; 2 = hind-limb paralysis or inability to turn to upright position; 3 = complete hind-limb paralysis and wasting; 4 = moribund or dead).

### Virus titers and cytokine determination

Virus titers within the brain were determined in clarified supernatants by plaque assay, using the murine DBT astrocytoma cell line as described previously [26]. Plaques were counted 48 hours after incubation at 37°C. Clarified supernatants were also used to measure IFN-γ by ELISA as described [22]. Briefly, 96 well plates were coated overnight at 4°C with 100 μl of 1 μg/ml of α-IFN-γ (R4-6A2; BD Biosciences, San Jose, CA, USA). Non-specific binding was blocked with 10% fetal calf serum in PBS for 1.5 h before the addition of IFN-γ recombinant cytokine standard (BD Biosciences) and samples. After a 2 h incubation at room temperature bound IFN-γ was detected using biotinylated α-IFN-γ antibody (XMG1.2, BD Biosciences) and avidin peroxidase followed by 3,3^′^,5,5^′^ tetramethylbenzidine (TMB Reagent Set; BD Biosciences) 1h later. Optical densities were read at 450 nm in a microplate reader (Model 680; Bio-Rad Laboratories, Hercules, CA, USA) and analyzed using Microplate Manager software (version 5.2; Bio-Rad Laboratories).

### Isolation of mononuclear cells

CNS-derived cells were isolated as described previously [20]. Briefly, brains or spinal cords from PBS-perfused mice (n = 3 to 6) were homogenized in ice-cold Tenbroeck tissue grinders in Dulbecco’s PBS. Homogenates were clarified by centrifugation at 400 *g* for 7 minutes, and the supernatants were collected and stored at −80°C for further analysis. Cell pellets were resuspended in RPMI supplemented with 25 mmol/l HEPES, adjusted to 30% Percoll (Pharmacia, Piscataway, NJ, USA) and underlaid with 1 ml of 70% Percoll. After centrifugation at 800 *g* for 30 minutes at 4°C, cells were recovered from the 30/70% interface, washed once, and resuspended in fluorescence-activated cell sorting (FACS) buffer. CNS-derived cell populations for PCR analysis were isolated from infected mice as described above. Cell suspensions from cervical lymph nodes (CLNs) were prepared from identical animals as previously described [20].

### Flow-cytometry analysis and fluorescence-activated cell sorting

Cells were incubated with mouse serum and rat α-mouse FcγIII/II mAb for 15 minutes on ice before staining. Expressionof cell surface markers was determined by incubation of cells with fluorescein isothiocyanate (FITC)-conjugated, phycoerythrin (PE)-conjugated, Peridinin Chlorophyll Protein Complex (PerCP) (PerCP)-conjugated, or allophycocyanin-conjugated mAbs specific for CD45 (30-F11), CD4 (L3T4), CD8 (53–6.7) CD44 (IM7), CD62L (MEL-14) (all BD Biosciences), PD-1 (RMP1-30; eBioScience San Diego, CA, USA) and F4/80 (CI:A3-1; Serotec, Raleigh, NC, USA) for 30 minutes on ice. Virus-specific CD8 T cells were identified using D^b^/S510 MHC class I tetramers (Beckman Coulter Inc., Fullerton, CA, USA) as described previously [20]. Stained cells were washed twice with FACS buffer and fixed in 2% paraformaldehyde. For intracellular detection of granzyme B or IFN-γ, the cells were stained for cell surface markers before permeabilization (Cytofix/Cytoperm Reagent; BD Biosciences) and staining with allophycocyanin-labeled α-granzyme B Ab (GB12, isotype-control mouse IgG1; Caltag Laboratories Burlingame, CA, USA) or α-IFN-γ Ab (BD Biosciences). A minimum of 2 × 10^5^ viable cells were stained and analyzed on a flow cytometer (FACS Calibur; BD, Mountain View, CA, USA). Data were analyzed using FlowJo software (Tree Star Inc., Ashland, OR, USA). CNS monocyte-derived CD45^hi^F4/80^+^ macrophages, CD45^lo^ microglia, and CD4 and CD8 T cells were purified from pooled brains (n = 6 to 8) using a cell sorter (FACSAria; BD). CD4CD44^hi^CD62L^lo^ (effector) and CD4CD44^lo^CD62L^hi^ (naive) cells were also purified from pooled CLNs. A minimum of 5 × 10^4^ cells were collected per pooled sample, and frozen in 400 μl Trizol reagent (Invitrogen, Carsbad, CA, USA) at −80°C for subsequent RNA extraction and PCR analysis as described previously [27].

Virus-specific IFN-γ production by CLN-derived CD8 T cells was evaluated after peptide stimulation. Briefly, 2 × 10^6^ CLN cells were cultured in the absence or presence of 1 μmol/l S510 peptide encompassing the H-2D^b^-restricted CD8 T-cell epitope in a total volume of 200 μl RPMI supplemented with 10% fetal calf serum for 5h at 37°C with a protein transport inhibitor (GolgiStop; BD Bioscience) at 1 μl/ml. After stimulation, cells were stained for surface expression of CD8, CD44, and CD62L, fixed, and then permeabilized to detect intracellular IFN-γ as recommended by the supplier (BD Biosciences).

### Histopathology

Spinal cords from PBS-perfused mice were fixed in 10% formalin and embedded in paraffin. In some experiments, the spinal cords were sectioned longitudinally, while in others they were cut into six segments from cervical to lumbar regions, and embedded together in paraffin. Cross-sections from individual mice were examined at each of the six levels. Demyelination was determined by staining 5 μm sections with Luxol fast blue (LFB), while axonal integrity was examined using the α-phosphorylated neurofilament mAb SMI-31 and the α-non-phosphorylated neurofilament mAb SMI-32 (Covance, Princeton, NJ). Viral nucleocapsid protein was detected by immunoperoxidase staining using the α-JHMV mouse mAb J.3.3 as the primary antibody, horse α-mouse as secondary antibody and 3,3^′^-diaminobenzidine (DAB) as substrate (Vectastain ABC kit; Vector Laboratories, Burlingame, CA, USA). Microglia and infiltrating macrophages were identified by immunoperoxidase staining using α-Mac-3 (BD Bioscience) as the primary antibody and rabbit α-rat as secondary antibody. With the exception of the LFB staining, all sections were counterstained with hematoxylin. Double immunoperoxidase staining for an oligodendroglial marker (rabbit α-glutathione S-transferase (α-GST; Enzo Life Sciences, Farmingdale, NY, USA) and viral antigen (J.3.3 mAb) was performed on paraffin embedded sections after antigen retrieval (Vector Laboratories);. Immunolabeling was identified using a commercial kit (Vectastain ABC) with DAB and Vector SG chromogens, respectively.

### Histologic imaging

High-resolution digital images were obtained using a slide scanner (ScanScope; Aperio, Vista, CA, USA) with a 20× lens objective and doubling lens. Images were viewed using ImageScope software (Aperio). For semi-quantitative analyses, sections were scored in a blinded fashion, and representative fields were identified based on the average score of all sections in each experimental group. For quantitative analyses (for example, Mac-3 and SMI-31 plus SMI-32 immunolocalization) images were analyzed using the ‘positive pixel count v9’ algorithm (Aperio) to obtain the percentage of positive pixels in representative fields of 5 mm^2^.

### PCR

RNA was extracted from FACS-purified cell populations frozen in 400 μl Trizol reagent (Invitrogen) as recommended by the supplier. DNA contamination was removed by treatment with DNase I (DNA-free kit; Ambion, Austin, TX, USA) for 30 minutes at 37°C, and cDNA was synthesized from RNA using reverse transcriptase (M-MLV; Invitrogen), oligo-dT primers and random primers (all Promega, Madison, WI, USA). Quantitative real-time PCR was performed using 4 μl of cDNA and SYBR Green (SYBR Green Master Mix; Applied Biosystes, Foster City, CA, USA) in triplicate on a PCR system (7500 Fast Real-Time PCR System; Applied Biosystems). PCR conditions were 10 minutes at 95°C followed by 40 cycles at 95°C for 15 seconds, 60°C for 30 seconds and 72°C for 30 seconds. Previously described primers were used for transcripts encoding glyceraldehyde 3-phosphate dehydrogenase (GAPDH), viral nucleocapsid, interleukin (IL)-10 and IL-21, and inducible nitric oxide synthase (iNOS) [15,28]. GAPDH, IL-2, IFN-γ and perforin mRNA levels were determined using gene expression arrays (Applied Biosystems) with a commercial master mix and primers (Universal Taqman Fast Master Mix and Taqman primers; both Applied Biosystems). PCR conditions were 20 seconds at 95°C followed by 40 cycles at 95°C for 3 seconds and 60°C for 30 seconds. Transcript levels were calculated relative to the housekeeping gene GAPDH using the formula:

$$2^{\left[\text{CT}\left(\text{GAPDH}\right)–\text{CT}\left(\text{target}\;\text{gene}\right)\right]}\;\times \;1000,$$

where CT represents the threshold cycle at which the fluorescent signal becomes significantly higher than that of the background.

### Statistical analysis

Results are expressed as the mean ± SEM for each group of mice. In all cases, *P*<0.05 was considered significant. Graphs were plotted and statistics assessed using GraphPad Prism software (version 3.0).

## Results

### B7-H1 deficiency does not compensate for the essential anti-viral role of CD4 T-cell help

**Figure 1:**
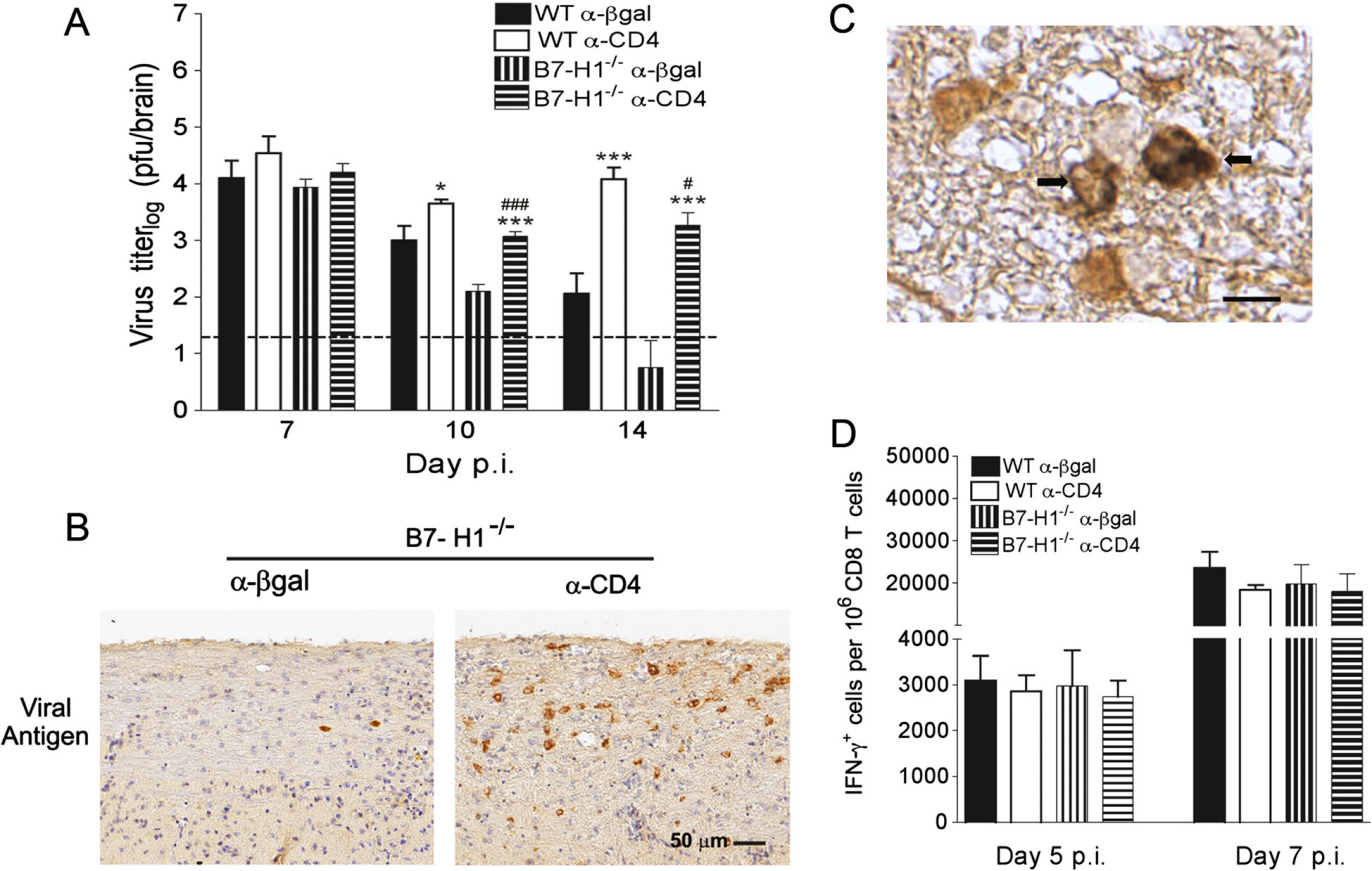
**Loss of central nervous system viral control in CD4-depleted B7-H1**^**−/−**^**mice.** Infected wild-type (WT) and B7-H1^−/−^ mice were treated with α-CD4 or control α-βgalactosidase (α-βgal) monoclonal antibody (mAb) at 4 and 6 days post-infection (p.i). (**A**) Virus titers in the brain determined by plaque assay expressed as mean ± SEM. Dashed line represents limit of detection. Significant differences between CD4-depleted and CD4-undepleted mice were determined by the unpaired *t*-test. * *P*<0.05 and *** *P*<0.001, respectively. Significant differences comparing CD4 depleted WT and B7-H1^−/−^ mice were determined by the unpaired *t*-test. ^#^*P*<0.05 and ^###^*P*<0.001, respectively. (**B**) Virus-infected cells detected in spinal-cord white-matter tracks using α-nucleocapsid mAb (J.3.3; brown) with hematoxylin counterstain at 10 days p.i. (**C**) Double immunohistochemical staining with rabbit α-glutathione S-transferase (α-GST) antibody labeling the oligodendrocytes (brown; DAB chromogen) and α-nucleocapsid J.3.3 mAb (blue/grey SG chromogen). Arrows point to virus-infected oligodendroglial cells (double-labeled), adjacent to non-infected oligodendroglial cells (labeled with DAB only). Magnification bar = 10 μm. (**D**) Frequencies of virus-specific interferon (IFN)-γ secreting CD8 T cells per 10^6^ CD8 T cells in CLNs at day 5 and 7 p.i. detected by flow cytometry after S510 peptide stimulation. Data are representative of two independent experiments with four mice per time point per experiment.

During sub-lethal JHMV infection of WT mice, CD4 T-cell help is required for efficient priming and expansion of virus-specific CD8 T cells in CLNs, and to support their effector function in the CNS [8]. To assess whether relief from B7-H1 inhibitory signals overcomes the local requirement of CD4 T cell help without affecting initial CD8 T cell expansion, CD4 T cell depletion was delayed until day 4 p.i. in B7-H1^−/−^ mice. Infectious virus levels were similar at day 7 p.i. in the brains of WT and B7-H1^−/−^ mice, irrespective of the presence or absence of CD4 T cells (Figure 1A). Furthermore, the virus was more effectively controlled in undepleted B7-H1^−/−^ relative to WT mice, confirming previous results [15,22]. In CD4-depleted WT mice, loss of viral control was evident at day 10 p.i., but was much more pronounced by day 14 p.i., relative to undepleted mice. By contrast, CD4-depleted B7-H1^−/−^ mice already harbored at least 10-fold more infectious virus at day 10 p.i. compared with undepleted B7-H1^−/−^ controls, and subsequently failed to control infectious virus (Figure 1A). Loss of viral control was thus accelerated in CD4 depleted B7-H1^−/−^ mice relative to the respective WT groups (Figure 1A). Nevertheless, relative to CD4-depleted WT mice, viral titers in CD4-depleted B7-H1^−/−^ mice were significantly lower at days 10 and 14 p.i., suggesting that unhelped CD8 T cells are still more functional in B7-H1^−/−^ than in WT mice. Consistent with the higher viral titers, the number of virus-infected cells exhibiting glial morphology, predominantly oligodendroglia, was increased in the spinal-cord white matter of CD4-depleted B7-H1^−/−^ mice (Figure 1B). Frequent infection of oligodendroglial cells was confirmed by double immunoperoxidase labeling with an oligodendroglial marker (α-GST) and anti-viral nucleocapsid antibody (J.3.3) (Figure 1C).

**Figure 2:**
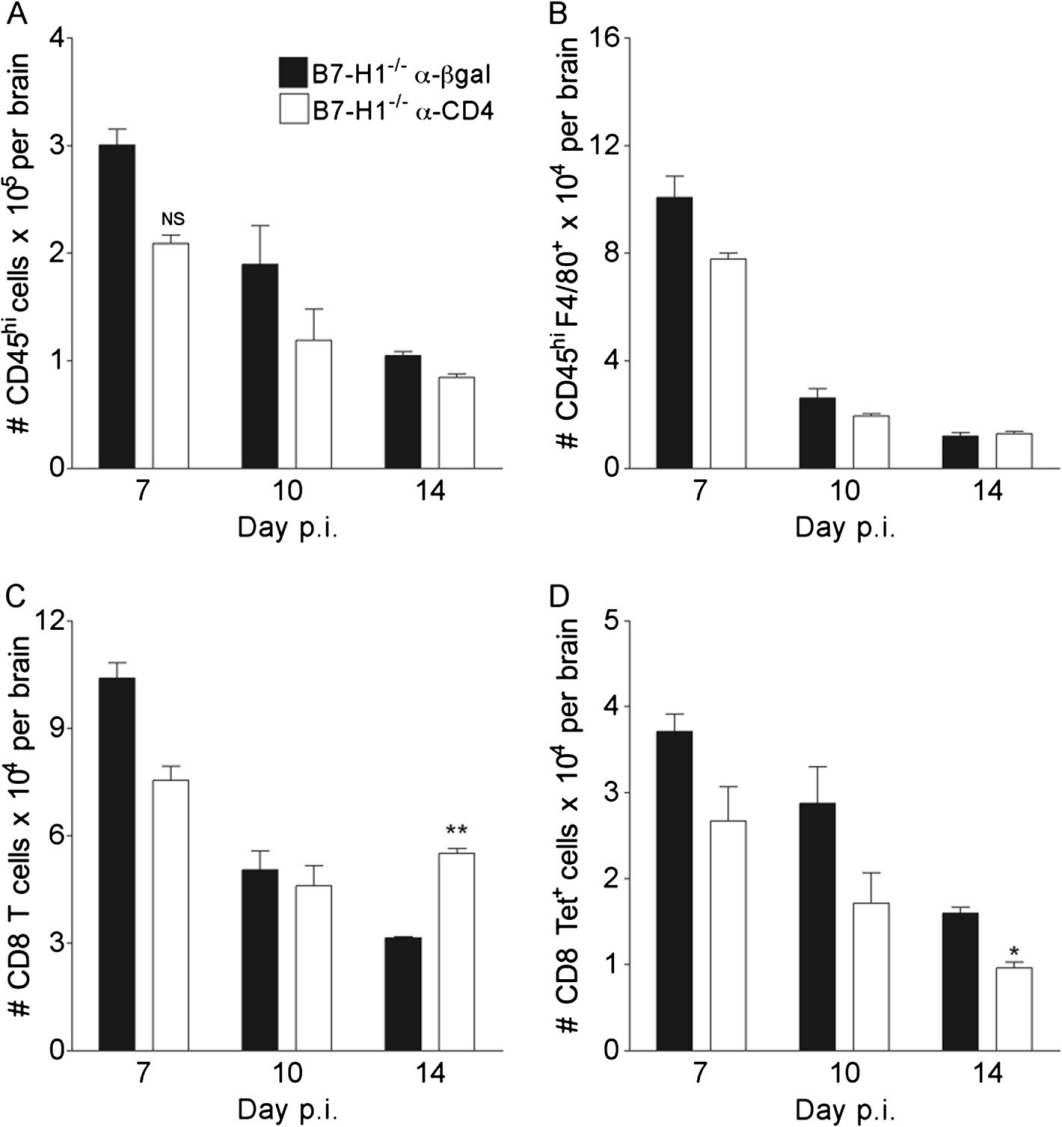
**Similar central nervous system recruitment of leukocytes in CD4 depleted B7-H1**^**−/− **^**mice.** B7-H1^−/−^ infected mice were treated with α-CD4 or control α-βgal monoclonal antibody at 4 and 6 days post-infection (p.i.). CNS-infiltrating cells were analyzed by flow cytometry from pooled brains. Numbers of (**A**) total CD45^hi^ CNS-infiltrating cells, (**B**) CD45^hi^F4/80^+^ macrophages, (**C**) CD8 T cells and (**D**) virus-specific D^b^/S510 tetramer^+^ CD8 T cells are shown. Data depict the mean ± SEM of two independent experiments with at least three mice per time point per experiment. Significant differences were determined by the unpaired *t*-test. * *P*<0.05 and ** *P*<0.005. NS = not significant.

Frequencies of IFN-γ-secreting CD8 T cells in the draining CLNs were not affected by the absence of B7-H1 or CD4 depletion in either WT or B7-H1^−/−^ mice (Figure 1D), supporting B7-H1-independent peripheral expansion [15] and intact peripheral CD4 T-cell help. Flow cytometry further showed that CD4 T-cell depletion did not overtly affect CNS cellular inflammation in B7-H1^−/−^ mice, as the numbers of CD45^hi^ CNS-infiltrating cells, F4/80-positive macrophages, total CD8 T cells, and virus-specific CD8 T cells were similar to those in undepleted B7-H1^−/−^ mice (Figure 2). Failure of virus control was thus not attributable to impaired peripheral expansion or CNS accumulation of virus-specific CD8 T cells. These results are similar to those seen in WT mice depleted of CD4 T cells subsequent to T-cell priming [8], and suggest that impaired viral control in CD4-depleted B7-H1^−/−^ mice results from diminished local CD8 T-cell function.

### Absence of B7-H1 does not rescue anti-viral function of unhelped CD8 T cells

**Figure 3:**
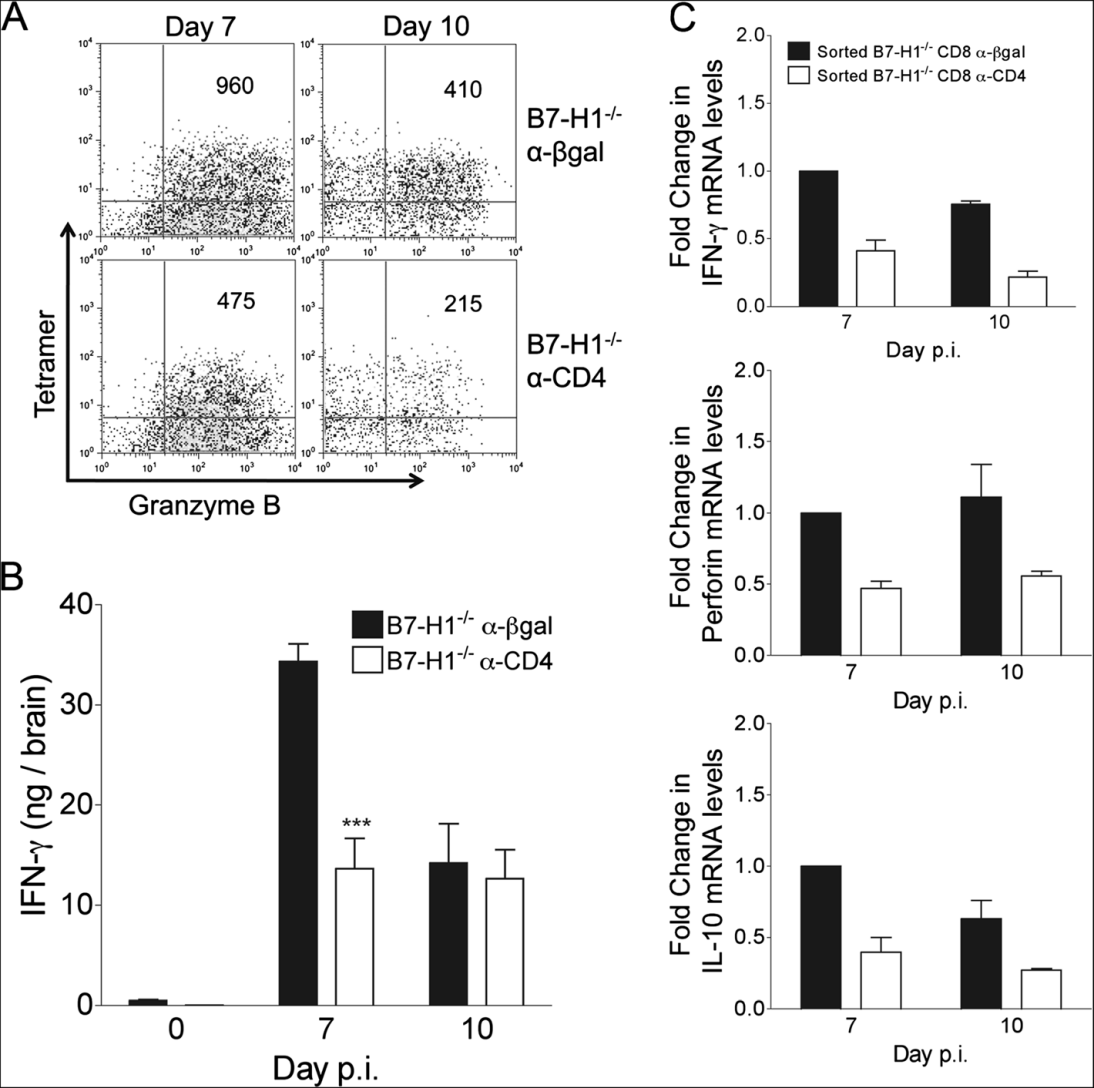
**Compromised CD8 effector function in the central nervous system of CD4-depleted B7-H1**^**−/− **^**mice.** Infected B7-H1^−/−^ mice were treated with α-CD4 or control α-βgalactosidase (α-βgal) monoclonal antibody at 4 and 6 days post-infection (p.i) (**A**) Pooled brain cells (n ≥ 4/group) isolated at days 7 and 10 p.i. were stained for CD8, D^b^/S510-specific T-cell receptor and intracellular granzyme B. Representative density plots gated on CD8 T cells depict α-granzyme B and Db/S510 tetramer staining. Mean fluorescence intensity (MFI) of granzyme B in D^b^/S510 tetramer^+^ CD8 T cells is shown in the upper right-hand quadrant. Data are representative of three independent experiments. (**B**) CNS interferon (IFN)-γ protein determined by ELISA at the indicated days p.i. Data represent the mean ± SEM (n ≥ 6/group) of two separate experiments. Significant differences were determined by the unpaired *t*-test. *** *P*<0.001. (**C**) Transcript levels of IFN-γ, perforin and interleukin (IL)-10 in CD8 T cells purified by fluorescence-activated cell sorting from the pooled brains of n = 6 to 8 mice collected at 7 and 10 days p.i. assessed by PCR. Transcript levels relative to GAPDH × 1000 are presented as fold change with levels from B7-H1^−/−^ α-βgal day 7 p.i. samples set to 1. Data depict the mean ± SEM of two independent experiments.

Virus-specific CD8 T cells derived from the CNS of CD4 T-cell sufficient or depleted B7-H1^−/−^ mice were assayed directly *ex vivo* without peptide stimulation for granzyme B expression as a marker for cytolytic capacity. Indeed, unhelped virus-specific CD8 T cells expressed lower levels of granzyme B compared with their helped counterparts at days 7 and 10 p.i. (Figure 3A). IFN-γ levels were also significantly reduced in the CNS of CD4-depleted B7-H1^−/−^ mice at day 7 p.i., but were similar to B7-H1^−/−^ controls by day 10 p.i. (Figure 3B). Impaired IFN-γ production specifically in CD8 T cells *in vivo* was supported by ~2 and 3-fold lower IFN-γ transcript levels specifically in CD8 T cells purified from the CNS of CD4-depleted mice relative to control B7-H1^−/−^ mice at days 7 and 10 p.i., respectively (Figure 3C). Similarly, transcript levels of perforin were reduced by around 2-fold (Figure 3C), correlating with diminished granzyme B expression in CD4-depleted B7-H1^−/−^ mice (Figure 3A). IL-10 expression was also assessed, as highly lytic CD8 T cells were recently associated with high IL-10 production [29]. IL-10 transcripts were decreased by around 2.5-fold in CD8 T cells from CD4-depleted B7-H1^−/−^ mice (Figure 3C), whereas tumor necrosis factor and IL-2 transcripts remained similar (data not shown), indicating that decreased function of B7-H1^−/−^ CD8 T cells in absence of CD4 T cells was not global. Therefore, blockade of PD-1:B7-H1 inhibitory signaling did not compensate for the crucial role of CD4 T-cell help in optimizing CD8 T-cell anti-viral function at the effector site.

### B7-H1 regulates CD4 T-cell function in the CNS

**Figure 4:**
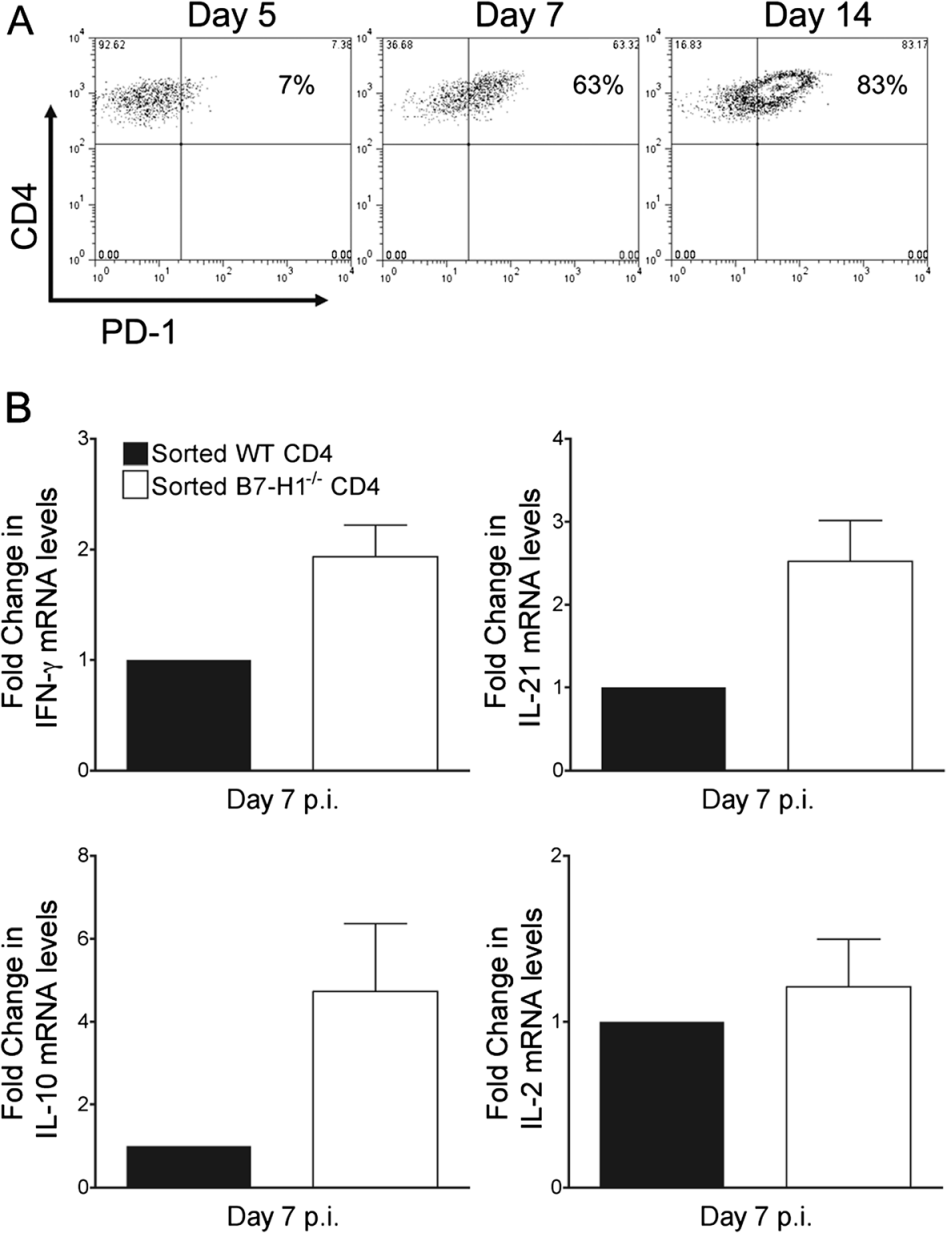
**B7-H1 negatively regulates CD4 T cell activity in the central nervous system.** (**A**) Representative density plots depicting PD-1 expression on CD4 T cells from spinal cords of infected wild-type (WT) mice at the indicated days post-infection (p.i).; numbers within plots represent percentage of PD-1-positive CD4 T cells. Data are representative of two independent experiments with three mice per time point. (**B**) Interferon (IFN)-γ, interleukin (IL)-2, IL-10, and IL-21 transcripts in purified CD4 T cells from WT or B7-H1^−/−^ brains at 7 days p.i. Transcript levels relative to GAPDH × 1000 are presented as fold change with levels from WT samples set at 1. Expression levels relative to GAPDH in WT samples were as follows: IFN-γ = 60; IL-10 = 55; IL-21 = 41; and IL-2 = 1.1. Data depict the mean ± SEM of two independent experiments.

**Figure 5:**
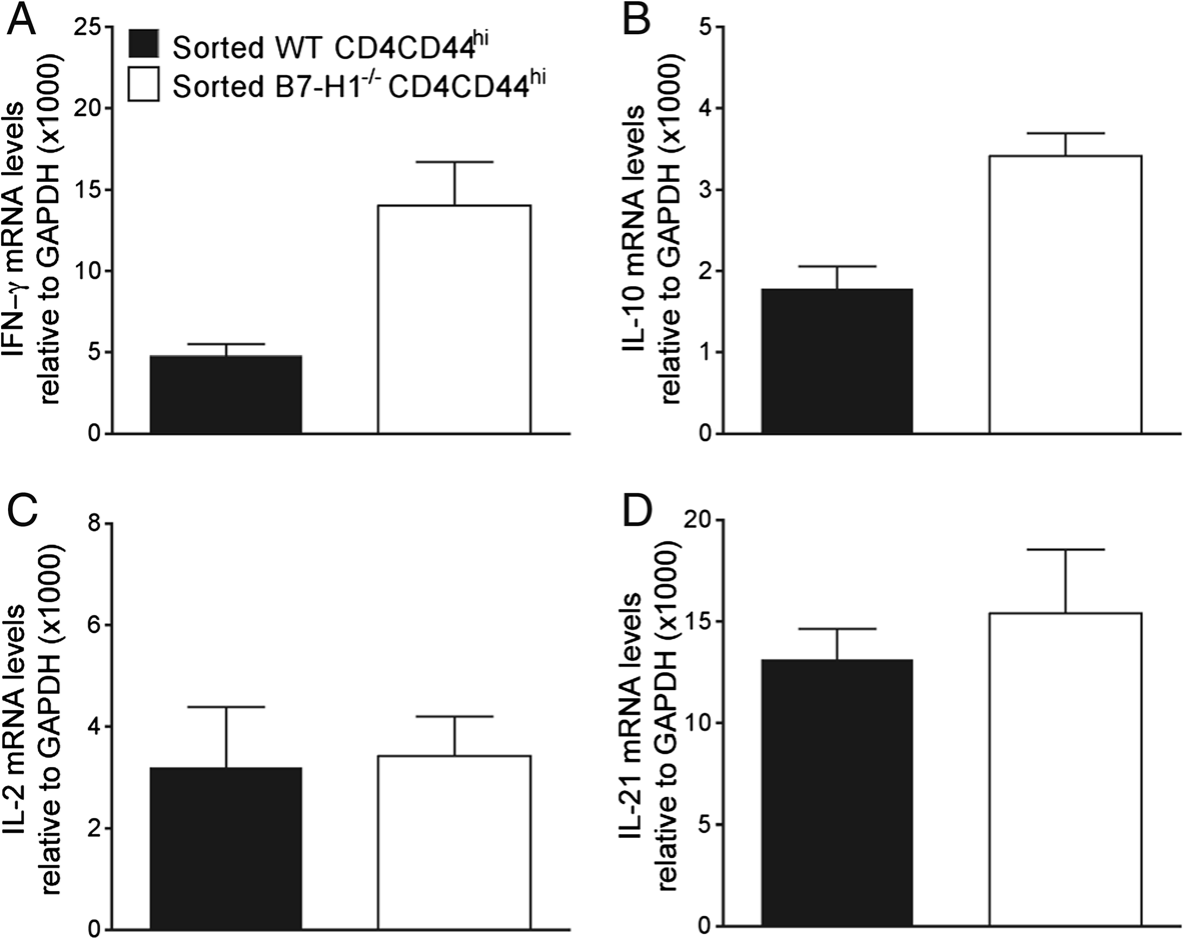
**B7-H1 dampens CD4 T-cell activity during priming.** (**A-D**) Relative transcript levels of (**A**) interferon (IFN)-γ, (**B**) interleukin (IL)-10, (**C**) IL-2 (**D**) and IL-21 in CD44^hi^CD62L^lo^ CD4 T cells purified by fluorescence-activated cell sorting from cervical lymph nodes of wild-type (WT) or B7-H1^−/−^ mice at 5 days post-infection. Transcript levels are relative to GAPDH × 1000. Data depict the mean ± SEM of two independent experiments.

Our original studies in B7-H1^−/−^ mice focused on CD8 T cells because oligodendrocytes are the major targets of infection and strongly upregulate B7-H1, coincident with MHC class I [22]. CD4 T cells were expected to have less pronounced effects relative to CD8 T cells, as infection of microglia and macrophages, constituting MHC class II-expressing APCs, is relatively sparse compared with oligodendrocytes [30]. Furthermore, B7-H1 expression is both modest and transient in microglia [22]. Nevertheless, comparable or higher expression of IFN-γ mRNA by CNS-infiltrating CD4 compared with CD8 T cells at the population level supported T-cell receptor-driven activity [15], and thus potential regulation by PD-1:B7-H1. Although less than 10% of CNS-derived CD4 T cells initially expressed PD-1 at day 5 p.i., this percentage rapidly increased to ~60% by day 7 p.i. and ~80% by day 14 p.i. (Figure 4A), showing that the vast majority of CD4 T cells infiltrating the CNS express PD-1 during control of the virus. To assess whether PD-1:B7-H1 interactions alter CD4 function, CD4 T cells were purified by FACS from the CNS of infected WT and B7-H1^−/−^ mice at day 7 p.i. IFN-γ mRNA levels were around 2-fold higher in CD4 T cells from B7-H1^−/−^ relative to WT mice (Figure 4B). Moreover, CD4 T cells from B7-H1^−/−^ mice had mRNA levels that were around 2.5-fold higher for IL-21 and 5-fold higher IL-10, compared with WT CD4 T cells (Figure 4B). However, the similar levels of IL-2 mRNA suggested that not all cytokines were affected by the absence of B7-H1. To assess whether cytokine-expression differences were imprinted during priming in the periphery or acquired within the CNS, activated CD44^hi^CD62L^lo^ and naïve CD44^lo^CD62L^hi^ CD4 T cells from the CLNs of infected WT and B7-H1^−/−^ mice were purified by FACS at day 5 p.i. (Figure 5). Cytokine mRNA levels for IFN-γ, IL-2, IL-10, and IL-21 were either undetectable or were below values of 0.1 in CD4 T cells expressing a naïve CD44^lo^CD62L^hi^ phenotype (data not shown). Similar to the CNS, IFN-γ levels in CD44^hi^CD62L^lo^ cells were around 3-fold higher in the absence of B7-H1 at 5 days p.i. A similar trend was apparent for IL-10 mRNA levels, which were increased by less than 2-fold in B7-H1^−/−^ CD4 T cells. By contrast, B7-H1 deficiency did not alter mRNA levels of IL-2 or IL-21 in activated CLN-derived CD4 T cells. Of note, IL-21, a positive regulator of CD8 T cells [31-35], was the only cytokine that was specifically higher in the CNS-derived but not in CLN-derived B7-H1^−/−^ CD4 T cells. These data suggest that PD-1 expression on CD4 T cells during JHMV infection negatively regulates CD4 T-cell effector activity both during initial expansion and in the CNS. Increased CNS effector function of CD8 T cells in infected B7-H1^−/−^ mice may thus not only be attributed to directly abrogated B7-H1 signaling, but also to enhanced local CD4 T cell help.

**Figure 6:**
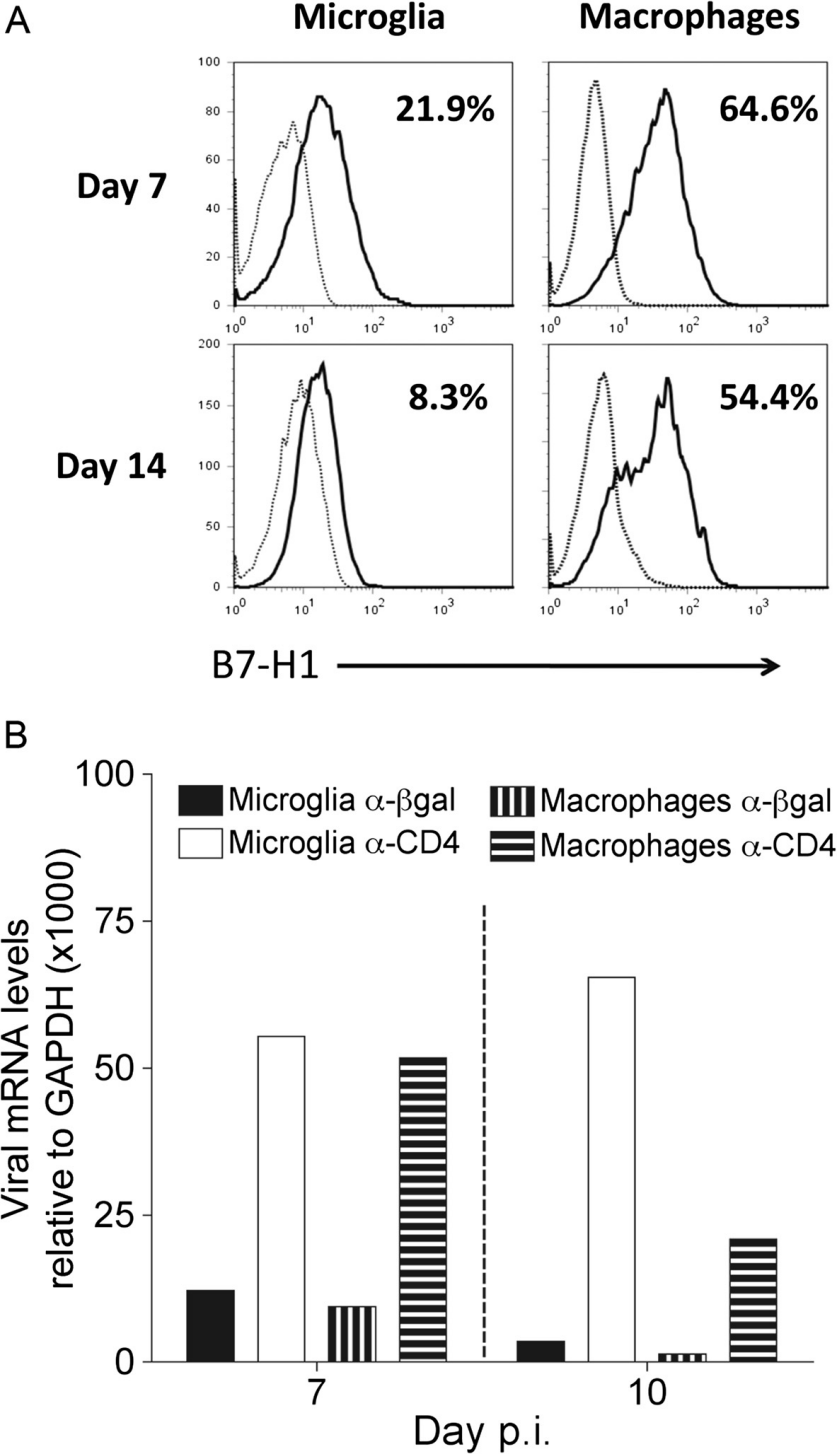
**Central nervous system-infiltrating macrophages display enhanced B7-H1 expression relative to microglia.** (**A**) B7-H1 expression on CD45^lo^ microglia and CD45^hi^F4/80^+^ macrophages from spinal cords of infected wild-type mice at the indicated days post-infection (p.i). Flow-cytometry histograms depict B7-H1 expression by solid lines, and isotype-control staining by dashed lines. Numbers represent percentages of B7-H1 positive cells relative to isotype controls. Data are representative of two independent experiments with three to five mice per timepoint. (**B**) Transcripts encoding viral nucleocapsid protein in microglia and macrophages purified from CD4-competent or CD4-depleted B7-H1^−/−^ mice (n = 6 to 8) at 7 and 10 days .p.i. Transcript levels are relative to GAPDH × 1000. Data are representative of two independent experiments with similar trends.

During the initial phase of JHMV infection, the virus replicates in both microglia and macrophages [27,30]. Both populations upregulate MHC class I and class II, and thus constitute targets capable of engaging CD4 and CD8 T cells within the CNS; astrocytes and oligodendrocytes fail to upregulate MHC class II [23,36]. Infected DCs are difficult to detect in the periphery [37,38] and have not been described within the CNS, to our knowledge. We therefore compared B7-H1 expression on CNS macrophages versus microglia. Microglia upregulated B7-H1 only modestly at 7 days p.i. (Figure 6A), confirming previous results [22]. By contrast, around 60% of macrophages expressed B7-H1 at 7 days p.i. (Figure 6A). Furthermore, whereas B7-H1 expression was transient on microglia, it was sustained on the majority of macrophages at day 14 p.i. The absence of B7-H1 on infected macrophages may thus directly underlie the enhanced CD4 T-cell effector activity in the CNS. To assess the potential effect of release from B7-H1 inhibition and CD4 depletion on viral replication in CD45^lo^ microglia and CD45^hi^F4/80^+^ macrophages, both cell populations were isolated from the CNS and analyzed for expression of viral mRNA. Transcripts encoding the viral nucleocapsid protein were higher in CD45^lo^ microglia and CNS CD45^hi^F4/80^+^ macrophages isolated from CD4-depleted mice compared with control B7-H1^−/−^ mice at both day 7 and day 10 p.i. (Figure 6B). Furthermore, both microglia and macrophages exhibited a reduction in viral mRNA between days 7 and 10 p.i. in undepleted mice, whereas only macrophages showed decreased viral mRNA in CD4 depleted mice between days 7 and 10 p.i.

**Figure 7:**
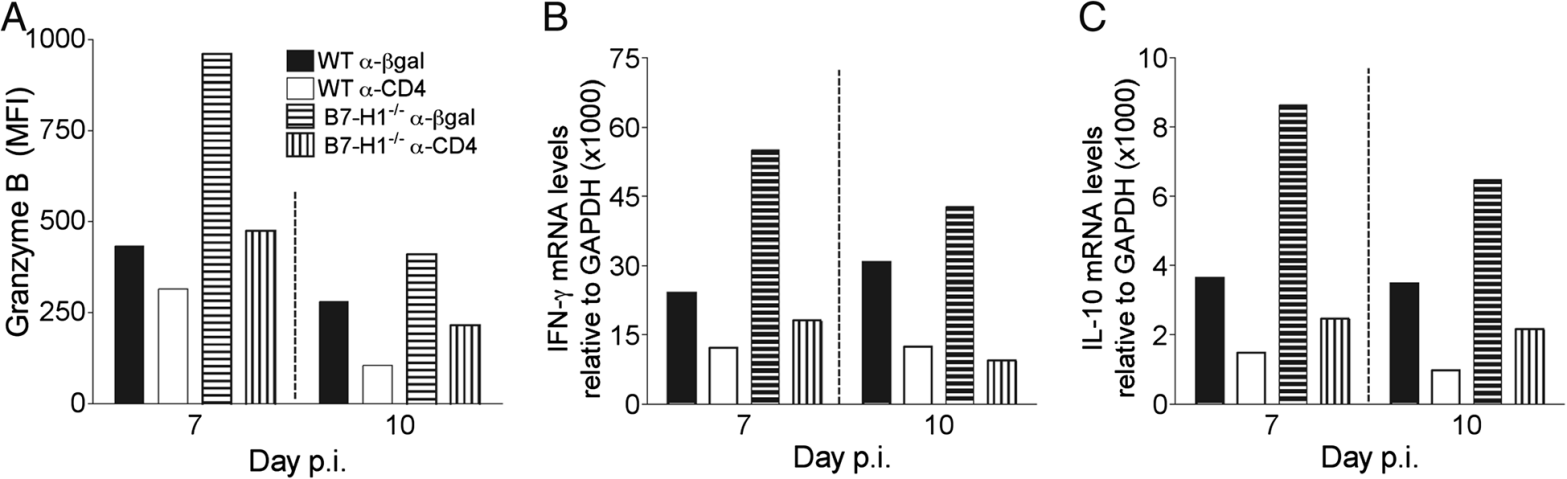
**B7-H1 deficiency directly increases CD8 T-cell activity in the absence of CD4 T cells.** Infected wild-type (WT) and B7-H1^−/−^ mice were treated with α-CD4 or control α-βgalactosidase (α-βgal) monoclonal antibody at 4 and 6 days post-infection (p.i). (**A**) Mean fluorescence intensity (MFI) of granzyme B expression by brain-derived virus-specific CD8 T cells (n ≥ 3/group) at days 7 and 10 p.i. Data are representative of two independent experiments with similar trends. (**B,C**) Relative transcript levels of (**B**) interferon (IFN)-γ and (**C**) interleukin (IL)-10 in CD8 T cells purified from brain at 7 and 10 days p.i. Transcript levels are relative to GAPDH × 1000. Data are representative of two independent experiments with similar trends.

These data support a contribution of CD4 T cells, either directly or indirectly, via enhancement of CD8 T-cell effector function in controlling virus in microglia/macrophages that express MHC class II. To reveal a direct effect of B7-H1 on CD8 T cells, effector functions in helped or unhelped CNS-derived B7-H1^−/−^ CD8 T cells were compared with their respective WT CD8 T-cell counterparts (Figure 7). In virus-specific CD8 T cells, the levels of granzyme B were comparable between unhelped B7-H1^−/−^ and helped WT populations at days 7 and 10 p.i. (Figure 7A). Moreover, granzyme B levels in unhelped B7-H1^−/−^ cells were higher than those in unhelped WT cells, but lower than those in helped B7-H1^−/−^ cells (Figure 7A). Notably, granzyme B levels correlated well with differences in viral load at day 10 p.i., especially in the comparison between WT mice and CD4-depleted B7-H1^−/−^ mice (Figure 1A). Transcript levels of IFN-γ in purified CD8 T cells from the CNS of CD4-depleted B7-H1^−/−^were similar to those in helped WT mice at day 7 p.i., but reduced by 60% at day 10 p.i. (Figure 7B). Similarly, IL-10 transcripts were equivalent in unhelped CD8 T cells from B7-H1^−/−^ and helped WT mice, supporting the notion that IL-10^+^ CD8 T cells are highly cytotoxic during JHMV infection [29]. Overall, these results suggest that unhelped *ex vivo* CD8 T-cell effector activity in B7-H1^−/−^ mice is comparable with helped WT CD8 T cells at day 7 p.i., and is only slightly diminished at day 10 p.i. Thus, B7-H1 blockade directly increases CD8 T-cell activity, even in the absence of CD4 T cells. However, the additional help provided by CD4 T cells in the absence of B7-H1 has an over-riding influence in both increasing CD8 T-cell activity early, and in prolonging function.

### CD4 T cells are mediators of exacerbated disease in B7-H1^−/−^ mice

**Figure 8:**
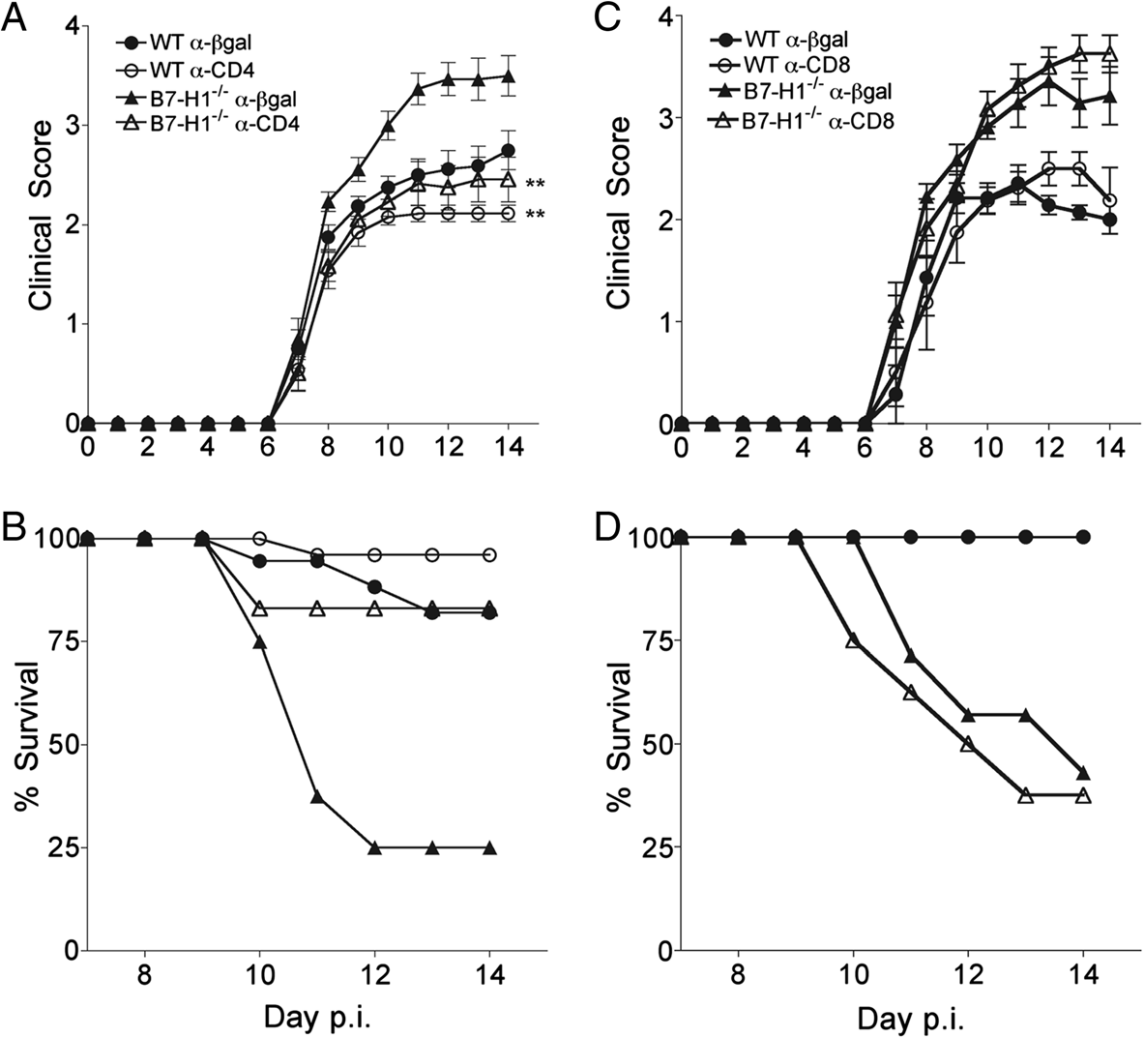
**CD4 depletion increases survival of B7-H1**^**−/− **^**mice.** Infected wild-type (WT) and B7-H1^−/−^ mice treated with α-CD4 or control α-βgalactosidase (α-βgal) monoclonal antibody (mAb) at 4 and 6 days post-infection (p.i) were monitored for (**A**) clinical symptoms and (**B**) survival. Significant differences in clinical symptoms comparing control and CD4-depleted WT and B7-H1^−/−^ groups, respectively, were determined by Wilcoxon matched pairs test. ** *P*<0.005. (**C**) Clinical symptoms and (**D**) survival of infected B7-H1^−/−^ mice treated with α-CD8 or control α-βgal mAb at days −2, 0 and 7p.i.

JHMV encephalomyelitis in B7-H1^−/−^ mice is associated with increased morbidity and axonal damage within the demyelinated lesions compared with WT mice, despite accelerated virus control [15]. These data were initially attributed to enhanced CD8 T-cell function mediated by release from B7-H1 interactions with infected oligodendrocytes. However, the absence of more severe demyelination posed a conundrum [15]. The present study demonstrates that CD4 T cells play a substantial role in contributing to enhanced IL-21 and IFN-γ production, which can be detrimental in the CNS [39,40]. Surprisingly, CD4 T-cell depletion in B7-H1^−/−^ mice lowered the severity of clinical disease substantially, and increased survival rates from around 30% in control B7-H1^−/−^ mice to around 80% (Figure 8A,B), despite higher viral load (Figure 1). Notably CD4-depleted B7-H1^−/−^ mice displayed slightly reduced clinical symptoms compared with CD4-competent WT mice, despite similar CD8 T-cell effector function in both groups (Figure 7). Similar, yet less prominent, results were seen in the comparison of CD4-depleted and control WT mice (Figure 8B). The extent to which enhanced disease severity and mortality in B7-H1^−/−^ mice is directly due to increased CD4 or CD8 T-cell activity is difficult to assess, as CD4 depletion also diminishes CD8 T-cell activity (Figure 7). However, the similar disease severity in infected WT mice (harboring helped CD8 T cells), and CD4 T cell-depleted B7-H1^−/−^ mice (harboring unhelped and uninhibited CD8 T cells), suggests that CD4 T cells primarily exacerbate CNS injury in B7-H1^−/−^ mice. This notion was supported by depleting B7-H1^−/−^ mice of CD8 T cells coincident with JHMV infection. Disease severity and mortality rates were similar to control B7-H1^−/−^ mice (Figure 8C, D), confirming CD4 T cells as prominent contributors to clinical disease during JHMV infection, if not regulated by B7-H1:PD-1 interactions.

**Figure 9:**
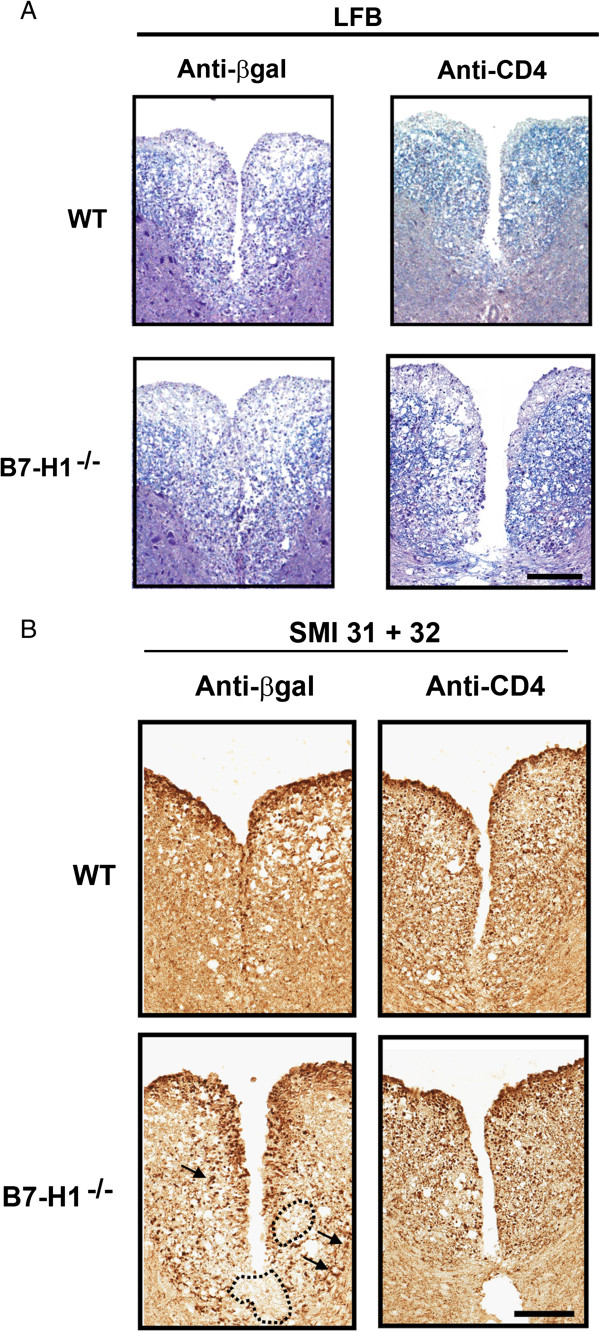
**Central nervous system pathology in CD4-depleted mice.** (**A**) Spinal cords at day 10 post-infection (p.i). stained with Luxol fast blue LFB (to identify areas of myelin loss) in wild-type (WT) and B7-H1^−/−^ mice show similar extent of demyelination after treatment with α-CD4 or control α-βgalactosidase (α-βgal) monoclonal antibody (mAb). Magnification bar = 100 μm. (**B**) Axonal integrity within demyelinated lesions visualized with α-SMI-31 plus α-SMI-32 Ab. Demyelinating lesions showed more extensive axonal damage in B7-H1^−/−^ mice, with larger areas of axonal loss (dotted outline) and increased numbers of swollen axons (arrows). After α-CD4 mAb treatment, lesions appeared to have reduced axonal loss and decreased numbers of swollen axons. Magnification bar = 100 μm.

**Figure 10:**
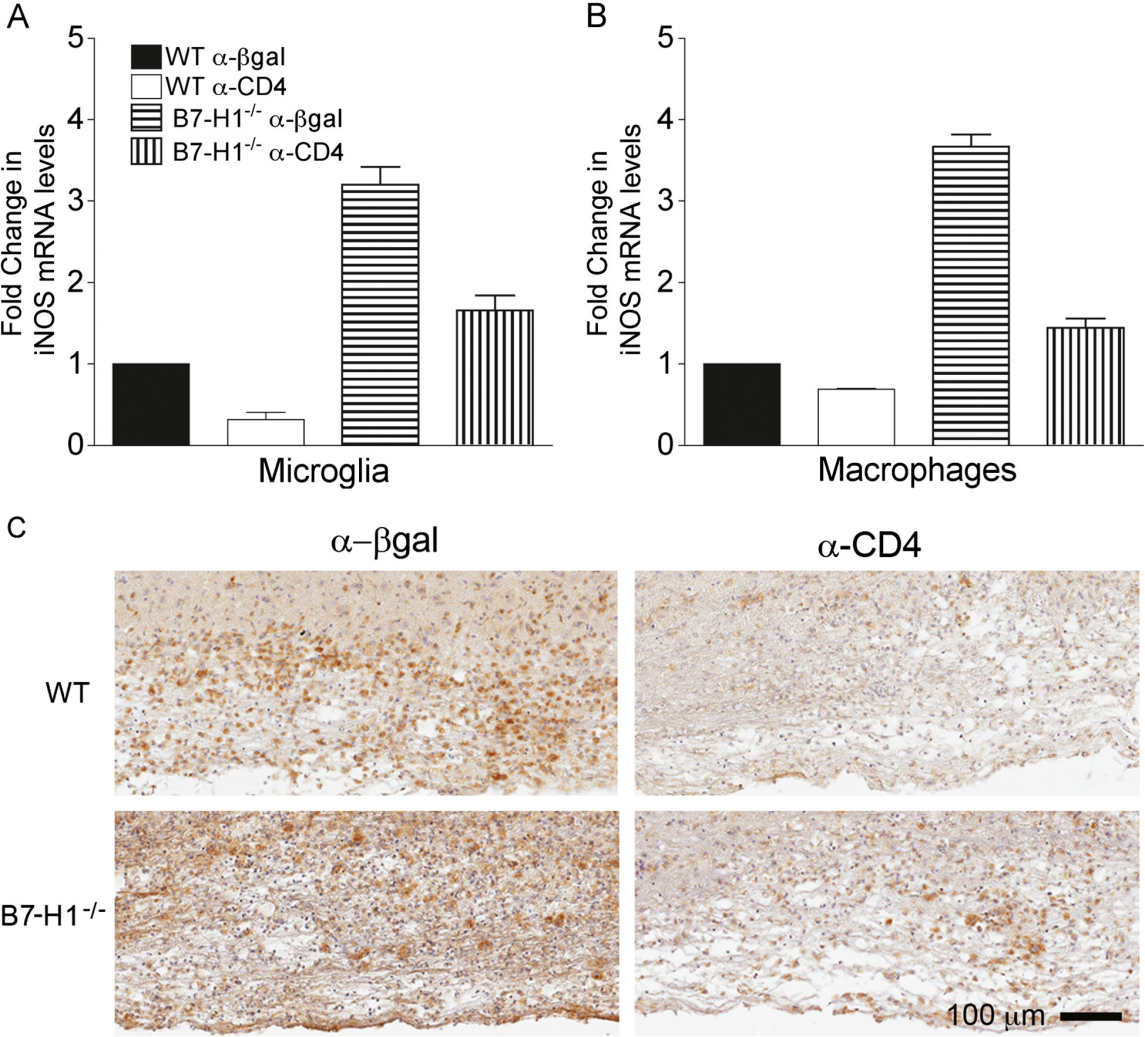
**Absence of CD4 T cells reduces microglia/macrophage activation.** Wild-type (WT) and B7-H1^−/−^ mice were treated with α-CD4 or control α-βgalactosidase (α-βgal) monoclonal antibody. (**A,B**) Fluorescence-activated cell sorting-purified CD45^lo^ microglia and CD45^hi^F4/80^+^ macrophages from pooled brains (n = 6 to 8) were assessed for transcript levels of inducible nitric oxide synthase (iNOS) relative to GAPDH × 1000 at 10 days post-infection (p.i). Transcript levels are presented as the fold change relative to levels from WT samples set to 1. Data depict the mean ± SEM of two independent experiments. (**C**) Microglia/macrophage activation assessed at day 10 p.i. by Mac-3 staining of spinal cords from WT (upper panels) and B7-H1^−/−^ (lower panels) mice that were depleted or sufficient in CD4 T cells.

Myelin loss and axonal integrity were assessed to determine whether the ameliorated disease in CD4 depleted B7-H1^−/−^ mice correlated with altered pathology. Myelin loss was monitored by LFB staining, while axonal integrity was assessed by dual staining with mAb SMI-31, specific for phosphorylated neurofilaments, and mAb SMI-32, specific for non-phosphorylated neurofilaments. Regardless of disease severity, CD4 T cells did not affect the overall size or number of focal demyelinating lesions in either WT or B7-H1^−/−^ mice (representative lesions shown in Figure 9A). Quantitative analysis of axonal staining (SMI-31 plus SMI-32) showed significantly less staining in B7-H1^−/−^ mice (18.1% axonal area) compared with WT controls (26.1% axonal area) indicating more axonal loss (*P*<0.02). B7-H1^−/−^ mice also exhibited increased numbers of swollen axons within the demyelinating lesions by qualitative observation (Figure 9B). Furthermore, the amount of axonal loss and number of swollen axons within the demyelinated lesion appeared to be reduced in CD4-depleted mice compared with their undepleted B7-H1^−/−^ counterparts; however, the difference in axonal loss did not reach statistical significance by quantitative analysis (Figure 9B). A potential mediator of increased neurotoxicity is IFN-γ-induced iNOS expression by microglia/macrophages. In CD4-competent B7-H1^−/−^ mice, enhanced morbidity and mortality is associated with sustained microglia/macrophage iNOS expression [15]. To assess how the decrease in IFN-γ within the CNS of CD4-depleted B7-H1^−/−^ mice affects iNOS expression, CD45^lo^ microglia and CD45^hi^F4/80^+^ macrophages were purified and their iNOS transcript levels compared. Expression of iNOS mRNA was considerably higher in both microglia and macrophages from infected CD4 competent B7-H1^−/−^ mice relative to their WT counterparts, with an increase of around 3-fold to 4-fold at day 10 p.i. (Figure 10A,B). Depletion of CD4 T cells in both WT and B7-H1^−/−^ mice reduced iNOS transcripts, consistent with the decreased levels of IFN-γ in the CNS (Figure 3). More importantly, iNOS expression in microglia and macrophages from CD4-depleted B7-H1^−/−^ mice were similar to those of the CD4-competent WT counterparts. These data suggest a correlation between diminished IFN-γ in CD4-depleted B7-H1^−/−^ mice and reduced microglia/macrophage activation with decreased axonal damage. Expression of Mac-3, a marker associated with myeloid cell activation, was assessed to further support the role of IFN-γ in microglia/macrophage activation (Figure 10C). Although the distribution of cells expressing Mac-3 within, and adjacent to, spinal-cord lesions was similar between all groups, differences were noted in the intensity of staining by qualitative observation. Therefore, we measured the percentage of Mac-3^+^ pixels using Aperio ScanScope software in multiple spinal-cord regions. Mac-3 staining was more intense in CD4-competent B7-H1^−/−^ mice (57% image area) compared with CD4- competent WT mice (43% image area; *P*<0.01), and was substantially reduced after CD4 depletion in both WT (33% image area; *P*<0.01) and B7-H1^−/−^(33% image area; *P*<0.03) mice (Figure 10C). These data support the notion that microglia/macrophage activation correlates with clinical severity.

## Discussion

Control of viral CNS infections is dependent on CD8 T-cell effector function in numerous rodent models [19,41-44]. Because many neurotropic viruses infect resident cells capable of upregulating MHC class I, but not class II molecules, effector mechanism are thought to result from direct target-cell/T-cell interactions. However, CD4 T cells play a supportive role for CD8 T cells that is less well understood, especially given the limited MHC class II expression, restricted to infiltrating APCs and microglia. Making matters more complex, effector functions by both T-cell subsets are regulated by inhibitory interactions in a cell type-specific manner. Specifically, B7-H1 blockade or deficiency enhances or reinvigorates CD8 T-cell function during prolonged antigen exposure in both visceral tissues and the CNS [3,45-48]. The extent to which potentially exacerbated CD4 T-cell function in the absence of B7-H1 inhibition influences CD8 T-cell activity and viral control is poorly explored. During gliatropic JHMV infection, CD8 T cells play a dominant role in controlling virus replication, but robust IFN-γ-mediated B7-H1 upregulation on oligodendrocytes, the prominent target of infection, delays viral control and contributes to persistence [15,22]. Accelerated viral control in the absence of B7-H1 thus provides a model to test whether CD4 T-cell help promoting CD8 T-cell activity in the CNS of WT mice is also a driving force for enhanced CD8 T cell activity in B7-H1^−/−^ mice.

The results clearly indicate that CD4 T cells are crucial in promoting the enhanced anti-viral CD8 T-cell response in B7-H1^−/−^ mice. Despite reduced CNS chemokine expression, likely resulting from reduced IFN-γ (data not shown), the absence of CD4 T cells did not affect initial CNS accumulation of CD8 T cells in B7-H1^−/−^ mice, similar to WT mice [8]. Reduced expression of perforin and IFN-γ transcripts and granzyme B by CNS-derived B7-H1^−/−^ unhelped CD8 T cells coincided with loss of CNS viral control at day 10 p.i. The sustained viral loads are likely responsible for the increased CNS numbers of total CD8 T cells at day 14 p.i. relative to control mice. Nevertheless, CD4-depleted B7-H1^−/−^ mice harbored at least 10-fold lower viral titers at days 10 and 14 p.i. relative to CD4-depleted WT mice, supporting higher activity of B7-H1^−/−^ CD8 T cells compared with WT CD8 T cells, regardless of CD4 help. This was substantiated by higher granzyme B levels in unhelped B7-H1^−/−^ CD8 T cells relative to unhelped WT CD8 T cells, and provides evidence that B7-H1 deficiency also directly contributed to enhanced CNS CD8 T-cell activity in the absence of CD4 T cells.

A direct inhibitory effect of B7-H1 engagement by CD4 T cells was suggested by the high expression of PD-1 on CNS-infiltrating CD4 T cells during JHMV infection in WT mice. B7-H1 mediated inhibition was indeed supported by increased IFN-γ, IL-21 and IL-10 transcript levels in CNS-derived B7-H1^−/−^ CD4 T cells relative to their WT counterparts. Increased CD4 T cell activity in the CNS of B7-H1^−/−^ mice has also been reported in the experimental allergic encephalomyelitis (EAE) model [49] and may indirectly contribute to increased anti-viral CD8 T-cell activity in the absence of B7-H1 in the CNS. Although increased IFN-γ and IL-10 mRNA in B7-H1^−/−^ relative to WT CD4 T cells was already imprinted during priming in CLNs, enhanced local CNS restimulation was supported by the specific increase of IL-21 transcripts and the overall 5-fold to 10-fold higher IFN-γ and IL-10 transcript levels in the CNS relative to CLN-derived B7-H1^−/−^ CD4 T-cell populations. Potential APCs which mediate B7-H1 suppressors of CD4 T cells within the JHMV-infected CNS are MHC class II positive macrophages, based on their strong B7-H1 expression compared with the modest and transient expression on microglia [22]. Although only few microglia/macrophages are infected, recent evidence demonstrates that CD4 T cells require only a few MHC class II-presenting cells to elicit IFN-γ secretion and long-range responsiveness *in vivo*[50]. However, although direct *in vivo* evidence for DC infection has been elusive, it cannot be excluded that viral antigen cross-presentation by DCs contributes to enhanced B7-H1^−/−^ CD4 T-cell effector function in the CNS. DCs presumably initiate T-cell priming within the CLNs, and the absence of constitutive B7-H1 expression on these APCs gives rise to enhanced activation of CLN-derived B7-H1^−/−^ CD4 T cells. Expression of IFN-γ and IL-10 mRNA was also greater in CLN-derived B7-H1^−/−^ CD8 T cells compared with their WT counterparts, although these differences were not reflected in the expansion of virus-specific CD8 T cells (data not shown). Overall, these results support early imprinting of B7-H1 on both CD8 and CD4 T-cell function during priming, which are further amplified by T cell restimulation within the CNS.

In addition to revealing a pronounced direct effect of B7-H1 on dampening CD4 T-cell activity, CD4 T-cell depletion indicated these lymphocytes as primary mediators of exacerbated disease in B7-H1^−/−^ mice, independent of increased viral load. This was supported by the inability of CD8 T-cell depletion to ameliorate disease in B7-H1^−/−^ mice. The implication that CD8 T cells alone might be candidates causing exacerbated disease ,based on more rapid viral control in B7-H1^−/−^ oligodendrocytes [15], which express MHC class I, but not class II, was thus not sustainable. Furthermore, whereas CD4 depletion in B7-H1^−/−^ mice ameliorated disease, despite impairing viral control, improvement of axonal damage did not reach statistical significance. These data, combined with similar demyelination, suggested additional affects of over-activated CD4 T cells on neuronal function, not necessarily reflected in histological readouts of neuronal integrity. Nevertheless, preservation of neurological function also correlates with axon sparing, regardless of demyelination during infection with Theiler’s murine encephalomyelitis virus [51,52]. The mechanisms underlying neuronal damage may be to due to misdirected T-cell activity or to secondary bystander effects. The absence of enhanced neuronal infection in CD4-competent B7-H1^−/−^ mice excluded a direct virus-mediated effect. Significantly decreased IFN-γ, reduced iNOS mRNA expression, and reduced Mac-3 reactivity in the CNS of CD4-depleted B7-H1^−/−^ mice supports a direct contribution of CD4-mediated microglia/macrophage activation to disease severity and mortality. Although macrophages can mediate demyelination in the absence of T cells during JHMV infection [53], apparent preservation of axonal integrity under these conditions suggests that CD4-mediated activation of the macrophages promotes axonal dysfunction. Involvement of blood-derived infiltrating macrophages in JHMV pathogenesis is also supported by decreased clinical severity in CCL2^−/−^ mice [54]. Similarly, inhibition of macrophage function in other experimental CNS diseases, such as EAE and encephalomyelitis caused by Theiler’s murine encephalomyelitis virus, ameliorates disease [55-57]. A contribution of iNOS as a detrimental effector molecule is consistent with the association between increased levels of iNOS expression and the pathological changes in other CNS disorders such as multiple sclerosis and its animal correlate EAE [58-62]. iNOS synthesizes the free radical nitric oxide (NO), which is implicated in pathologic processes due to its cytotoxicity at high concentrations and the destructive molecules generated from NO such as peroxynitrite [63-66] . Although iNOS does not seem to contribute directly to JHMV pathogenesis [67], it cannot be excluded that increased levels of NO or its products in B7-H1^−/−^ mice have deleterious local effects in the CNS.

## Conclusions

This study demonstrates that CD4 T-cell helper functions contribute significantly to enhanced CD8 T-cell activity in B7-H1^−/−^ mice, in addition to the direct relief of CD8 T cells from PD-1:B7-H1 inhibitory signaling within the CNS. PD-1:B7-H1 blockade is thus insufficient to overcome CD4 T cell helper function to CD8 T cells during JHMV infection. Moreover, CD4 T cells are prominent contributors to bystander pathology during JHMV infection if not regulated by B7-H1:PD-1 interactions.

## Abbreviations

Ab: Antibody; APC: Antigen-presenting cell; βgal: βgalactosidase; CLN: Cervical lymph node; CNS: Central nervous system; DAB: diaminobenzidine; DC: Dendritic cell; FACS: Fluorescence-activated cell sorting; FITC: Fluorescein isothiocyanate; GADPH: glyceraldehyde 3-phosphate dehydrogenase; IFN: Interferon; IL: Interleukin; iNOS: Inducible nitric oxide synthase; JHMV: Gliatropic JHM strain of mouse hepatitis virus; LFB: Luxol fast blue; MHC: Major histocompatibility complex; NO: Nitric oxide; PBS: Phosphate-buffered saline; PE: Phycoerythrin; PerCP: Peridinin chlorophyll protein complex; p.i.: Post-infection; PD-1: Programmed death-1; PFU: Plaque-forming unit; WT: Wild-type.

## Competing interests

The authors declare no competing financial interests.

## Authors’ contributions

TP designed and performed the experiment, collected and analyzed data, and wrote the manuscript; SAS interpreted data and wrote the manuscript; DRH analyzed and interpreted data; and CCB designed the research, provided materials, interpreted data, and wrote the manuscript. All authors read and approved the final manuscript.
